# Supplementary material for: Altered microRNA Transcriptome in Cultured Human Liver Cells upon Infection with Ebola Virus
Source: Int J Mol Sci. 2021 Apr 6;22(7):3792. doi: 10.3390/ijms22073792 (PMC8038836; doi:10.3390/ijms22073792)
Supplement: Supplementary file 1 [file ijms-22-03792-s001.zip › Supplementary_File/C_ GO_Analysis_Results/16-30nt_go_Mayinga-24h-Huh7_vs_Control-24h-Huh7_down.mature_mirna_targets/MF_FoldEnrichmentDotPlot.pdf]

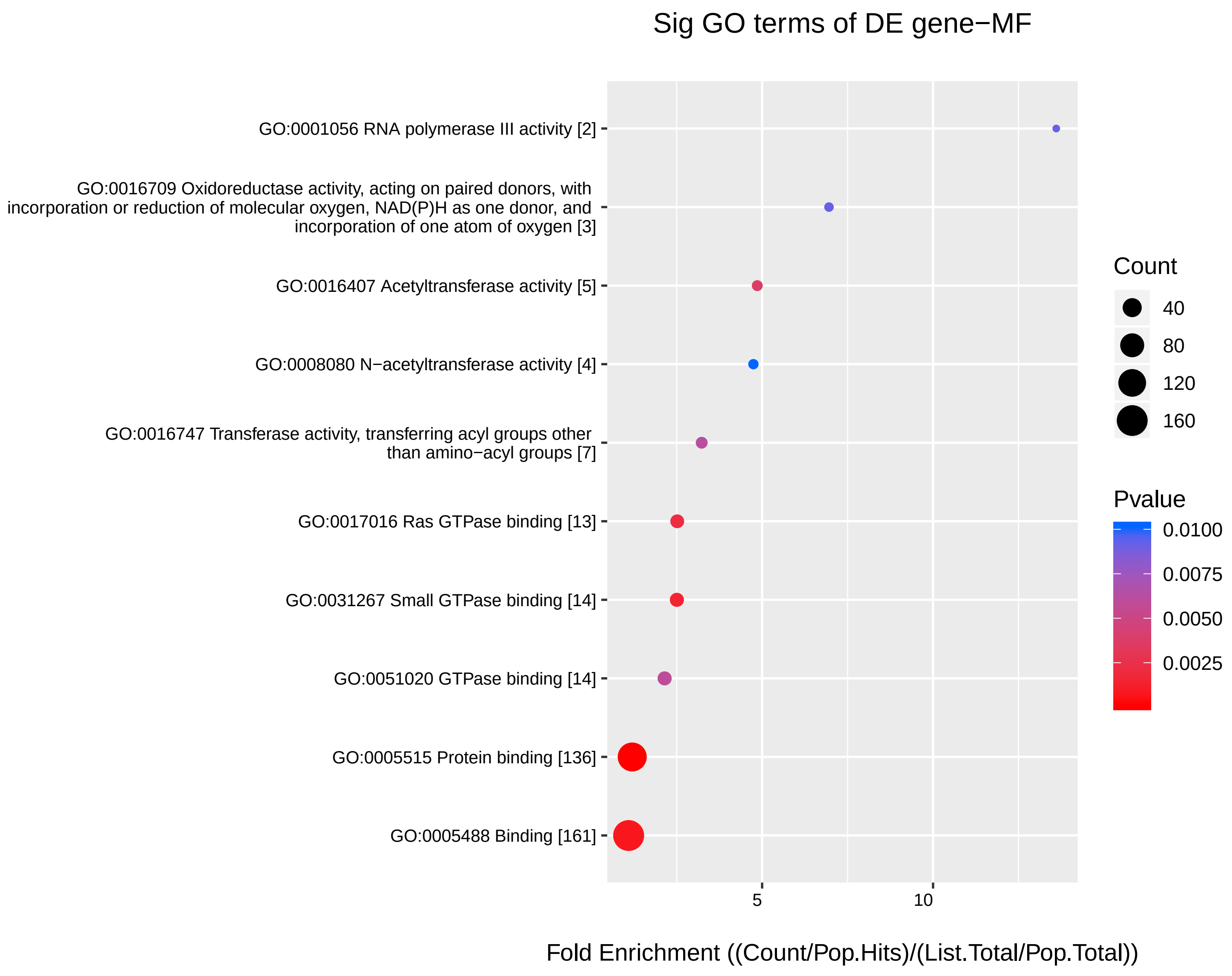
Sig GO terms of DE gene−MF
GO:0001056 RNA polymerase III activity [2]
GO:0016709 Oxidoreductase activity, acting on paired donors, with incorporation or reduction of molecular oxygen, NAD(P)H as one donor, and incorporation of one atom of oxygen [3]
GO:0016407 Acetyltransferase activity [5]
GO:0008080 N−acetyltransferase activity [4]
GO:0016747 Transferase activity, transferring acyl groups other than amino−acyl groups [7]
GO:0017016 Ras GTPase binding [13]
GO:0031267 Small GTPase binding [14]
GO:0051020 GTPase binding [14]
GO:0005515 Protein binding [136]
GO:0005488 Binding [161]
5
10
Fold Enrichment ((Count/Pop.Hits)/(List.Total/Pop.Total))
Count
40
80
120
160
Pvalue
0.0100
0.0075
0.0050
0.0025
